# Non-Canonical Notch Signaling in Cancer and Immunity

**DOI:** 10.3389/fonc.2014.00345

**Published:** 2014-12-04

**Authors:** Furkan Ayaz, Barbara A. Osborne

**Affiliations:** ^1^Program in Molecular and Cellular Biology, University of Massachusetts, Amherst, MA, USA; ^2^Department of Veterinary and Animal Sciences, University of Massachusetts, Amherst, MA, USA

**Keywords:** Notch, non-canonical, T lymphocytes, cancer, signal transduction

## Abstract

Canonical Notch signaling is initiated by γ-secretase-mediated cleavage of the Notch receptor, leading to the release of the active intra-cellular domain of Notch that migrates to the nucleus and interacts with RBP-Jκ, resulting in the activation of downstream target genes. While canonical Notch signaling is well known to play an active role in several steps during development as well in multiple cell fate decisions, recent evidence from both invertebrate and vertebrate systems indicates that non-canonical, RBP-Jκ-independent signaling is important in several cellular processes including oncogenesis and activation of T lymphocytes. These observations raise the possibility that, through an understanding of non-canonical Notch signaling, novel strategies for inhibiting Notch signaling may prove useful in the design of therapies targeted to block aberrant Notch activity. In this mini-review, we will examine the current data demonstrating a non-canonical role for Notch signaling in both cancer and the immune system and suggest a better understanding of non-canonical signaling may reveal novel strategies to block Notch signaling in disease.

## Introduction

Notch is a trans-membrane protein with four family members (Notch 1–4). Canonical Notch signaling is initiated by interaction of the Notch protein with a cell bound ligand (Delta-like 1, 3, 4 or Jagged 1, 2) and results in cleavage of Notch, initially by ADAM10 and ADAM17 proteases, followed by cleavage by the gamma-secretase complex (1–3). At the completion of this process, the Notch intra-cellular domain (NICD) translocates into the nucleus and interacts with RBPJκ/CSL, a transcriptional repressor. Upon interaction with NICD, RBPJκ/CSL is converted into a potent transcriptional activator of downstream target genes (4).

However, more recent studies reveal the existence of several other modes of Notch signaling generally referred to as non-canonical Notch signaling (5). Interestingly, many instances of non-canonical signaling are associated with potentially pathological conditions including cancer and activation of the immune system while many normal cellular processes require canonical Notch signaling. For example, it is likely that early development in the mammalian embryo requires canonical Notch signaling since deletion of RBP-Jκ mimics’ deletion of Notch1 (6, 7). Several other physiological processes, such as maintenance of the intestinal epithelium, also require canonical Notch signaling (8). Therefore, it is possible that blockade of non-canonical Notch signaling may provide opportunities to inhibit some instances of pathological Notch signaling leaving many other normal physiological processes intact. However, since non-canonical Notch signaling is not as well characterized as the canonical signaling pathway, more in-depth inquiries in this area are likely to reveal potential new targets to manipulate non-canonical Notch signaling. Below, we describe a number of instances where non-canonical Notch signaling is associated with cancer or abnormal immune function and we propose that a better understanding of these pathways may uncover new opportunities for therapeutic approaches.

## Non-Canonical Notch Signaling in Cancer

The first indication for a role of Notch pathway in oncogenesis came from Aster et al. (9) and Pear et al. (10) in T-cell acute lymphoblastic leukemia (T-ALL) in which chromosomal translocation of the *Notch1* gene was identified as a cause of T-cell oncogenesis. In later reports, the Notch pathway has been associated with tumorigenesis and cancer progression in the other cancers including breast, ovarian, cervical, lung, prostate carcinomas, gliomas, and mesotheliomas (6, 9–16). It is well documented that Notch signaling regulates proliferation, differentiation, and survival of tumor cells (17, 18) and also is reported to maintain the stem cell-like characteristics of cancer stem cells (19–21). Notch is also required for further progression of differentiated cancer cells by regulating metabolism, survival, and transcription in these cells. In addition to its role in tumorigenesis, Notch has also been reported to act as a tumor suppressor in certain cell types such as skin epithelium (22). This observation makes it quite clear that an understanding of individual Notch signaling pathways is important for the rational therapeutic manipulation of Notch.

Inhibition of γ-secretase does not block all Notch-related functions in tumor cells, suggesting a role for non-canonical Notch signaling in transformed cells (6, 8, 9, 11–13, 16). Additionally, transformation of baby rat kidney cells through cooperation between the adenoviral E1A protein and NICD does not require the RBPJκ/CSL binding domain of NICD, suggesting that transformation in this system may be non-canonical. However, non-canonical nuclear localization of NICD was still required for oncogenesis (23, 24).

### Non-canonical Notch signaling in leukemia

Studies by Vacca et al. (25) suggest that non-canonical Notch3 signaling regulates T-cell development and leukemia through activation of the NF-κB pathway. In their transgenic mouse model, Notch3 overexpression, specifically in T cells, led to the development of leukemia (25). This group showed that increased Notch3 expression enabled constitutive activation of NF-κB and demonstrated that Notch3 interacts with IKKα to maintain NF-κB activity (25).

In human myelogenous leukemia cells, Notch1 directly interacts with the transcription factor, YY1, to drive expression of the oncogenic transcription factor *c-myc* independently of CSL (26). In HPV-driven human cervical cancer, non-canonical Notch signaling enables oncogenesis, independently of CSL, via PI3K pathway (27). However, little is known about how non-canonical Notch signaling drives transformation in these situations.

### Non-canonical Notch signaling in the mammary gland

Raafat et al. (28), using conditional RBPJκ knockout mice, revealed that non-canonical Notch4 signaling is involved in mammary gland tumorigenesis, whereas canonical Notch4 was required for the development of mammary glands (28). This differential regulation provides an attractive opportunity for targeting non-canonical Notch signaling to dampen oncogenesis while enabling intact tissue homeostasis and development to occur via canonical Notch signaling. Another study suggests an RBPJκ-independent role for Notch4 signaling in the survival of endothelial cell lines (29). Furthermore, during breast cancer progression, Notch signaling plays a role in epithelial transformation independent of CSL (30). These studies further emphasize the importance of non-canonical Notch signaling in breast cancer cell survival and progression.

Additionally, in breast cancer cell lines, non-canonical Notch signaling is known to regulate IL-6 expression, and IL-6, in turn, acts on tumor cells to further increase their oncogenic potential. In this study, cytoplasmic NICD interaction with the NF-κB pathway induced IL-6 expression (31). These studies, in addition to those reported above in leukemic T cells (25), support a role for non-canonical Notch signaling via NF-κB pathway in oncogenesis.

### Non-canonical Notch signaling in apoptosis and metabolism

Non-canonical Notch signaling also is implicated in the regulation of metabolism in tumor cells. Recent studies from Perumalsamy et al. (32) demonstrated that non-nuclear, either cytoplasmic or membrane tethered, NICD blocks starvation-induced apoptosis in HeLa, a cervical cancer cell line. This group also showed that nuclear retention of NICD abrogates its anti-apoptotic activity thus demonstrating that, in their system, Notch controls apoptosis via a non-canonical, cytosolic pathway. Their data further suggest that non-canonical Notch regulation of apoptosis occurs through the mTOR–Rictor pathway (32). Another study linking Notch to metabolism examined the role of Notch in regulating neuronal stem cells (33). This report demonstrated an interaction between Notch and the PTEN-induced kinase 1 (PINK1) and provides evidence that the Notch/PINK1 interaction influences mitochondrial function and activates the mTORC2/Akt pathway. This non-canonical Notch signaling induced proliferation and tumor stem cell maintenance through mitochondrial and metabolic pathways. Additionally, in this study, the authors observed localization of full length Notch1 on the mitochondrial membrane further linking at least some forms of non-canonical Notch signaling to the mitochondria (33).

Non-canonical Notch signaling is known to regulate hypoxic pathways in transformed human cell lines (34). In this study, it was shown that NICD sequesters a negative regulator of HIF-1α resulting in increased protein levels of HIF-1α and, in turn, increasing its downstream effects (34). Since hypoxia and metabolic changes are hallmarks of tumor tissue, the emerging role of non-canonical signaling in these pathways implies that there will be increased recognition of non-canonical Notch signaling mechanisms and cross-talk with other important pathways in a variety of tumor settings (35).

## Non-Canonical Notch Signaling in the Immune System

Notch signaling regulates some lineage decisions of hematopoietic cells (36, 37), and enables generation of T cells at the expense of B and myeloid cells in the early stages of hematopoietic cell development. At later time points, Notch plays a key role in the survival, proliferation, and differentiation of T cells. Notch signaling also regulates the development of some innate lymphoid cells, marginal zone B cells from precursor B cells, megakaryocytes, and cytotoxic (CD8^+^) T-cell lymphocytes (CTLs) (38–43). Thus, as discussed more extensively below, non-canonical Notch signaling is involved in the development and function of several types of immune cells.

### Non-canonical Notch signaling in T-cell activation and differentiation

Notch is important in driving the differentiation of naïve CD4^+^ T cells into specific T helper (Th) subsets and targeting Notch signaling in Th cells provides the opportunity for immune modulation. Studies in our lab demonstrate that GSI treatment significantly reduces Th1, Th17, and induced Treg (iTreg) polarization (44, 45). Studies by other labs using different methods to block Notch signaling showed that Th2 polarization is also driven by Notch signaling (46, 47). We demonstrated a significant decrease in Th1 and iTreg differentiation in conditional Notch1 knockout Th cells and through the use of conditional RBPJκ knockout T cells, revealed that Notch regulates Th cell differentiation into different Th cell fates independent of RBPJκ and hence is non-canonical. Furthermore, our data showed that both activation and proliferation of CD4^+^ T cells are not impaired by conditional deletion of RBPJκ (45). Thus, CD4^+^ T-cell activation, proliferation, and differentiation all require non-canonical Notch signaling, and recent data from our lab suggest Notch, in conjunction with NF-κB, and regulate this non-canonical signaling in CD4^+^ T cells. (45).

The possibility that non-canonical Notch signaling may occur through activation of NF-κB is not surprising since links between Notch and NF-κB have been documented by several groups including our own (48, 49). In cells of the immune system, Notch3 in collaboration with NF-κB is reported to cooperatively regulate FoxP3 expression (50). Additionally, we recently reported that Notch1 can initiate NF-κB activation via cytosolic interactions with components of the T-cell signalosome. In particular, cytosolic Notch1 drives the formation of the CARMA1, BCL10, and MALT1 (CBM) complex that is essential for NF-κB activation in T cells. These data demonstrated that cytosolic, rather than nuclear, Notch1 drives CBM complex formation emphasizing the non-canonical role of Notch1 in this process (49).

### Non-canonical Notch signaling in T-cell metabolism

In addition to Notch signaling through NF-κB, non-canonical Notch signaling is implicated in T-cell metabolism and cell survival. Upon lymphocyte activation, there is an immense change in the metabolic activity of T cells to enable the production of building blocks for cell division and growth as well as ATP production. This metabolic switch is closely linked with cell survival. As described above, Perumalsamy et al. (32) described a link between non-canonical Notch signaling and the mTORC2-Akt cascade (32). In this report, they also provide evidence that cell survival of activated T cells is regulated by the interaction of cytoplasmic or membrane tethered NICD with the mTORC2-Akt cascade and this may also be involved in cell metabolism (32). The same group had previously demonstrated that interaction of Notch1 and kinases involved in early T-cell activation (PI3K and p56^lck^) regulates an anti-apoptotic program in T cells through Akt signaling (51). Interestingly, another group has demonstrated mitochondrial localization of full length Notch1 protein in neuronal cells providing additional evidence in another system for non-canonical Notch signaling in metabolism and cell survival (33).

### A role for ligand-independent activation of Notch

As described above, canonical Notch signaling begins with the interaction between Notch and its ligand and this interaction catalyzes a series of events leading to cleavage and release of NICD. A conundrum in the immune system is the observation by our group and others, that activation of the T-cell receptor leads to rapid release of NICD (52, 53) and this may occur in the absence of ligand. While the mechanism of Notch activation through the TCR is poorly understood, a possible hint to this process is suggested by studies of *Drosophila* immune cells showing NICD can be generated independently of ligand interaction and this is dependent upon HIF-1α-mediated stabilization of NICD (52). Since ligand-independent Notch activation can occur through HIF-1α in *Drosophila* immune cells, it is tempting to speculate that this also may occur in mammals (54). The observed ligand-independent activation in immune cells of *Drosophila* may possibly be physiologically relevant in mammals. There is no evidence that Th cells express Notch ligands in the circulation while trafficking to effector sites after initial priming in lymph nodes and continuous Notch signaling could provide survival and differentiation signals in the circulation; however, this remains speculative.

A role for membrane tethered Notch is found in dendritic cells, a cell found at the nexus of the innate and adaptive immune system. According to one recent study, in dendritic cells, membrane bound Notch activates PI3 kinase. This non-canonical Notch signaling regulates the production of the immune suppressive cytokine, IL10, by dendritic cells in response to LPS (55).

Notch signaling in the immune system, while required for normal immune function, also is linked to several diseases of the immune system (38–41, 56–60). Aberrant Notch signaling is implicated in several autoimmune diseases including bone marrow failure, experimental autoimmune encephalomyelitis, rheumatoid arthritis, and type 1 diabetes (59, 61–63). Many of these diseases are caused, at least in part, by auto-reactive T cells. Since we know that Th1 and Th17 cell differentiation requires non-canonical Notch signaling, it is reasonable to envision a therapeutic strategy that would block non-canonical Notch signaling perhaps leaving intact other Notch signaling pathways important for normal function of other cells and tissues. However, to achieve such a goal, it is essential that we better understand the various pathways of non-canonical Notch signaling.

## Summary and Future Perspectives

**Figure 1:**
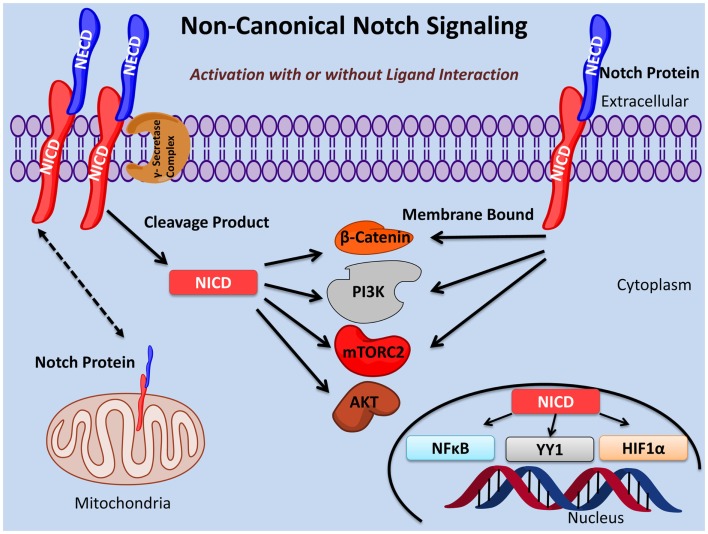
**Non-canonical Notch signaling pathways**. Non-canonical Notch signaling may occur either dependent or independent of ligand interaction. Additionally, non-canonical Notch signaling may be γ-secretase dependent or independent with the latter exerting its function as membrane bound Notch. Non-canonical Notch signaling is independent of CSL/RBPJκ and, instead, interacts with PI3K, mTORC2, AKT, Wnt, NFκB, YY1, or HIF-1α pathways at either the cytoplasmic and/or nuclear levels. Non-canonical Notch signaling regulates cell survival, metabolism, and differentiation through interaction with these pathways in many important biological processes including immunity and cancer.

Notch signaling plays an important role in the fine tuning of oncogenesis and immunity as well as many other essential biological processes. Here, we provide evidence for three types of non-canonical Notch signaling: (i) γ-secretase regulated activation of the Notch pathway that occurs independently of ligand interaction; (ii) NICD activity independent of RBPJκ/CSL; (iii) membrane bound Notch signaling in the absence of cleavage by the γ-secretase complex and, in some cases, independent of ligand interaction (Figure 1).

The studies described in this review emphasize the role of non-canonical Notch signaling in both cancer and the immune system. These studies highlight the various strategies employed by non-canonical Notch to drive a multitude of biological effects. A clear appreciation of both canonical and non-canonical Notch will deepen our understanding of the basic biology of Notch signaling. Inhibitors are available for many of the signaling pathways involved in non-canonical Notch signaling (NF-κB, PI3K, AKT, mTOR, HIF-1α, and β-catenin) and, in several instances, these inhibitors have passed through clinical trials. Therefore, it is possible to consider combination therapies where one of these inhibitors, perhaps in conjunction with reduced doses of a gamma-secretase inhibitor, might prove efficacious. For example, in T cell-mediated autoimmunity, where we know Notch and NF-kB cooperate to mediate aberrant Th1 activation (64), one might use a combination of gamma-secretase inhibition with curcumin, a neutracuetical known to inhibit NF-kB signaling (65). Thus, in the near future, it will be possible to test the possibility that combination therapy, using Notch inhibitors together with inhibitors of these other pathways, may be more efficacious in the treatment of diseases regulated by Notch. Careful delineation of Notch signaling pathways both in normal cells and tissues as well as in auto-reactive or oncogenic situations may produce a deeper and more nuanced understanding of this important signaling pathway and hence provide a roadmap for the identification of new and novel drug targets useful in the treatment of disease.

## Conflict of Interest Statement

The authors declare that the research was conducted in the absence of any commercial or financial relationships that could be construed as a potential conflict of interest.
